# Increased Mortality Associated with Well-Water Arsenic Exposure in Inner Mongolia, China

**DOI:** 10.3390/ijerph6031107

**Published:** 2009-03-16

**Authors:** Timothy J. Wade, Yajuan Xia, Kegong Wu, Yanhong Li, Zhixiong Ning, X Chris Le, Xiufen Lu, Yong Feng, Xingzhou He, Judy L. Mumford

**Affiliations:** 1 US Environmental Protection Agency, National Health and Environmental Effects Research Laboratory, Chapel Hill, North Carolina, USA; E-mail: mumford.judy@epa.gov; 2 Inner Mongolia Center for Endemic Disease Control and Research, Huhhot, Inner Mongolia, China; E-mails: yajxia@sohu.com (Y.X.); wkg1959@163.com (K.W.); nmgliyh@163.com (Y.L.); 3 Ba Men Anti Epidemic Station, Bayingnormen, Inner Mongolia, China; E-mail: bsjkxd@163.com; 4 University of Alberta, Department of Public Health Sciences, Edmonton, Canada; E-mails: xc.le@ualberta.ca (X.C.L); xlu@ualberta.ca (X.L.); 5 Hangjin Hou Centers for Disease Control, Hangjin Hou, Inner Mongolia, China; E-mail: Bingyu33@sina.com; 6 Chinese Academy of Preventative Medicine, Institute of Environmental Health &Engineering, Beijing, China; E-mail: He_2007@188.com

**Keywords:** Arsenic, drinking water, China, Inner Mongolia, mortality, heart disease, cancer

## Abstract

We conducted a retrospective mortality study in an Inner Mongolian village exposed to well water contaminated by arsenic since the 1980s. Deaths occurring between January 1, 1997 and December 1, 2004 were classified according to underlying cause and water samples from household wells were tested for total arsenic. Heart disease mortality was associated with arsenic exposure, and the association strengthened with time exposed to the water source. Cancer mortality and all-cause mortality were associated with well-water arsenic exposure among those exposed 10–20 years. This is the first study to document increased arsenic-associated mortality in the Bayingnormen region of Inner Mongolia.

## 1. Introduction

Worldwide, millions of people are at risk from the adverse effects of arsenic exposure through drinking water. Studies in Taiwan, Argentina, the United States and Chile have documented increased bladder, lung and skin cancer mortality as a result of arsenic exposure [1, 2, 3]. Chronic arsenic exposure has also been associated with non-cancer health effects including vascular and cardiovascular effects, diabetes, peripheral neuropathies, and adverse birth outcomes [4, 5, 6, 7, 8].

The Bayingnormen (Ba Men) region located on the Hetao Plain, north of the Huang He (Yellow) River in western Inner Mongolia, China, is affected by chronic arsenic exposure from contaminated well water especially in the three counties of Hangjin Hou, Lin He and Wu Yuan. In the late 1970s, the primary source of drinking water shifted away from large shallow wells to deeper artesian wells, which were often contaminated by naturally occurring arsenic. Characteristic arsenic-induced skin lesions were reported in the region in the early 1990s [9].

Ba Men is a rich agricultural region and most residents of the region are farmers. Most households receive their water from a hand pump well located adjacent to the household compound. Because of a lack of access to other types of water this well is their primary source of water for drinking, cooking, bathing and other household uses. Well water appears to be the primary source of arsenic exposure in the Ba Men region. Ma [9] reported that drinking water was the only significant source of arsenic exposure, that arsenic containing pesticides had not been used, and that samples from soil, food and surface water met or were lower than China national standards.

Several studies have documented the prevalence of arsenicosis as well as other adverse health effects associated with arsenic exposure from this region [9, 10, 11, 12, 13]. However, few systematic investigations of excess mortality have been conducted. We conducted a retrospective mortality study of one arsenic-exposed village in the Ba Men region, Shahai village, located in Hangjin Hou. While we recognized that the number of deaths from this relatively small village (approximately 12,000 residents) would not be enough to examine cause-specific mortality for specific conditions (for example, bladder cancer mortality) in relation to arsenic exposure, we anticipated sufficient cases to evaluate broader causes of mortality such as all-cancer mortality, and heart disease mortality. Our specific goals were to:

1. Identify deaths that occurred in the village between January 1, 1997 and December 1, 2004 and classify each according to underlying cause of death;
2. Evaluate the relationship between mortality rate and arsenic exposure from the primary water source.

## 2. Methods

### 2.1. Census and Mortality Classification

Cause of death is not systematically recorded in Ba Men. To classify causes of death, we conducted a complete census of the village. Each family provided names and demographic characteristics of all family members residing in the household between January 1, 1997 and December 1, 2004, including those who moved away and those who died.

Interviewers from the Inner Mongolia Center for Endemic Disease Control and Research (IMCEDCR), and the Ba Men Anti Epidemic Station visited each family in the village and interviewed an adult household member during November and December 2004. Two interviewers visited each household, and at least one was a medical professional (nurse or physician). The interview consisted of a census of household members (including those who had moved), health and mortality assessments. The interviews included questions about demographic characteristics for each person living in the household since January 1, 1997, including date of birth, sex, ethnicity, occupation, smoking habits (current smoker, quit, and years smoked) alcohol use (every day, sometimes or never) and a detailed residence history. The residence history consisted of a description of the water source (hand pump well, large mouth well, community well, or other), and the dates and location of the residence for each family member for the past 30 years.

We obtained information about each death occurring in the household since January 1, 1997. The deceased’s closest living relative provided a description of the conditions surrounding the death, and details about any health conditions afflicting the deceased. This verbal description was recorded to accurately preserve the description for cause of death classification. All medical records relating to the deceased including doctor’s notes, diagnoses, and medication information were collected. Study personnel also obtained the names and locations of any physicians, hospitals or clinics visited by the deceased. These physicians and clinics were contacted, additional existing medical records were obtained, and whenever possible, the attending physician was interviewed. A team of medical experts including a pathologist, a nosologist with training in the International Classification of Diseases, 10th Revision (ICD-10) [14], and a medical epidemiologist evaluated the evidence available and coded each underlying cause of death according to the ICD-10 system. When additional expertise was required for classification, specialists in other medical fields were consulted. The medical team was unaware of and blinded to the arsenic exposure levels from the water source. For analysis and description, we grouped ICD-10 codes according to the 113 leading causes of mortality defined by the National Center for Health Statistics [15]. The number deaths occurring in the village during the period were confirmed with local health officials.

Research staff at the Ba Men Anti-Epidemic Station double-entered all data in a MS Access database, pre-programmed with logic checks and restricted entry to reduce data entry errors. Discrepancies were identified and corrected by referring to the original survey form and if necessary, contacting the respondent for additional information and clarification.

### 2.2. Water Collection and Arsenic Analysis

At the time of interview, we collected approximately 40 ml of water in 50 ml polypropylene test tubes at the household’s primary source of water. We previously tested the collection tubes washing them with water containing acid (nitric acid) and tested them for arsenic concentrations, which were below detection limit. During arsenic analysis of the samples, we also included the negative controls using the same type of tube containing known arsenic-free water. The arsenic concentrations in these negative controls were below detection limit. After collection, the samples were stored at −20°C and then delivered on ice via air to University of Alberta in Edmonton, Canada for analysis. We measured total arsenic in water samples using inductively coupled plasma mass spectrometry (ICPMS) as described previously [13, 16]. The detection limit for ICPMS is 0.1 *μ*g/L. For some households (N=141), water samples were analyzed by the IMCEDCR using Atomic Fluorescence (AF, detection limit=0.4 *μ*g/L).

We assigned arsenic exposures based on the measured arsenic value from the resident’s well, shared well, or community well. For those results below the detection limit, we assigned one-half the detection limit as the exposure value.

### 2.3. Statistical Methods

Based on estimates of mortality rates and the size of the village, we initially calculated 80% power to observe an Incidence Rate Ratio (IRR) of 2 for a 50 *μ*g/L increase in arsenic exposure for heart disease mortality, and between 70% and 80% statistical power for total cancer and cerbrovascular disease (stroke) mortality.

We used Poisson regression to model mortality rates and to estimate the Incidence Rate Ratio (IRR) as a function of person-years of arsenic exposure. To account for the potential time-lag between arsenic exposure and observed population mortality rate, we restricted the analysis to those who had been exposed at the same water source since 2000 (approximately 5 years), 1995 (approximately 10 years), 1990 (approximately 15 years) and 1985 (approximately 20 years).

We controlled for age in years (continuous), sex (male versus female), education level (coded as an ordinal variable, 1 through 5, ranging from incomplete primary school through college degree) current smoking status (smoker versus non-smoker), and current alcohol consumption (yes or no). These covariates were considered for multivariate models based on their marginal association with arsenic, or their known or suspected association with the causes of mortality under study. We compared the change in the arsenic coefficient from the fully adjusted models, to models adjusting for just age and sex (reduced model), and an unadjusted model. Because the fully adjusted model usually indicated at least some confounding (based on a change in the arsenic coefficient), we reported results of the fully adjusted models. In these models, arsenic was the primary exposure of interest and was modeled as a continuous variable. We also evaluated arsenic effects by grouping exposures into five arsenic categories: 0–5, 5.1–20, 20.1–100, 100.1–300, and greater than 300 *μ*g/L and created corresponding indicator variables. For comparison purposes, we calculated age-adjusted mortality rates with direct standardization [17], using the United States Standard Population from 2000 [15].

All regression models used robust standard error calculations to correct for the clustered household sample [18]. We used Stata 9.2 for all statistical analysis [19].

## 3. Results

### 3.1. Mortality

**Figure 1.**
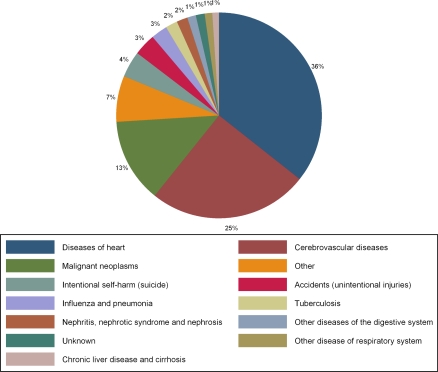
Leading causes of mortality among residents of Shahai Village, Inner Mongolia.

A total of 572 incident deaths were identified in the period between January 1, 1997 and December 1, 2004. Characteristics of the deceased residents are shown in Table 1. The mean age of death was 66 years of age. Deceased women were older than deceased men at the time of death (68 years of age vs. 64 years of age, p=0.03). Leading causes of death are summarized in Figure 1. Diseases of the heart were the leading causes of mortality, accounting for 206, or 36% of the 572 deaths. Of these, 87 were due to acute myocardial infarction or cardiac arrest. Cerebrovascular diseases, including stroke, were the next most frequently reported cause of death making up 25% of the deaths, followed by malignant neoplasms (13%), suicide (4%), and accidents (3%). Lung cancer was the most frequent type of cancer, causing 19 deaths, followed by stomach cancer (11 deaths). Cause of death could not be classified for five deaths. Forty deaths classified as ”Other” occurred fewer than four times and included causes classified as: legal intervention, homicide, septicemia, trauma, poisoning and diabetes.

Residents contributed a total of 78,251 person-years of time since January 1, 1997, with a mean of 6.1 person-years. The crude mortality rate was 731 deaths per 100,000 person years, 814 per 100 000 person-years among men and 646 per 100,000 person years among women. The age-adjusted mortality rates were 1096 per 100,000 person-years among all residents, and 1203 and 946 per 100,000 person years among males and females, respectively. For comparison, the 2003 age-adjusted mortality rate of the United States population was 831.2 among all subjects, and 991.7 and 705.4 per 100,000 among males and females respectively. Using the World Health Organization standard population for direct adjustment [20], the age-adjusted mortality rate in Shahai village for this time period was 720.6 per 100 000 person-years compared to 842.5 per 100 000 for the 2002 China population [21]. This lower mortality rate may reflect the generally good health status of the population region which has been noted previously [13, 22].

### 3.2. Arsenic Exposure

Among both living and deceased residents, total arsenic in water samples from current primary source of water ranged from below detection to 637.7 *μ*g/L, with mean exposure of 38.0 *μ*g/L (median 21 *μ*g/L). Arsenic exposures among deceased residents are summarized in Table 1. Arsenic exposures for a total of 52 households (included 10 deceased) could not be assigned due to either an unavailable or damaged water sample, or because the deceased moved out of the village prior to death. An additional 141 households were assigned arsenic values using data provided by the IMCEDCR. A total of 65 households had results for water arsenic below the detection limit (46 with the ICPMS method and 19 from the AF method) and were assigned arsenic exposures one-half the detection limit. Fifty of the 562 deceased residents for whom arsenic exposures were available (9%) were exposed to well water arsenic above 100 *μ*g/L and nearly three quarters used a hand-pump well for their primary source of water (Table 1). Among both deceased and living subjects, residents reported using their current water source a mean of 9.9 years, with deceased residents using their current water source an average of 11.6 years.

### 3.3. Mortality and Arsenic Exposure

Results from multivariate Poisson regression models summarizing the effect of a 50 *μ*g/L increase in arsenic on all-cause mortality, heart disease mortality, all-cancer mortality and cerebrovascular disease mortality are summarized in Table 2.

Among all residents, there was a significant association between heart disease mortality and arsenic exposure. A 50 *μ*g/L increase in arsenic was associated with a 12% increase in heart disease mortality (IRR=1.12, p=0.03). The risk of heart disease mortality increased as time exposed to the water source increased. Among residents exposed to the same well since before 2000, a 50 *μ*g/L increase in arsenic exposure was associated with a 16% increase heart disease mortality rate. The association peaked among residents exposed since before 1990. Among these residents, a 50 *μ*g/L increase in arsenic was associated with a 24% increase in heart disease mortality rate.

Although there were fewer cancer deaths, there was also some evidence of an association between arsenic exposure and total cancer mortality. A significant trend was evident among residents exposed since before 1995 (IRR=1.18, p=0.048), and was slightly stronger among residents exposed since before 1995 and 1990 (Table 2).

A 50 *μ*g/L increase in arsenic exposure was also associated with a 12% and 15% increase in all-cause mortality among residents exposed since before 1990 and 1985, respectively (Table 2).

Crude mortality rates and adjusted IRRs for residents exposed since before 1990 and since before 1995 are shown in Table 3 and Table 4. Although there were few deaths due to cancer above 300 *μ*g/L, heart disease mortality rates increased across categories of arsenic exposure and both cancer and all cause mortality peaked in the most highly exposed groups. In the categorical analysis, a decreased mortality due to stroke was observed for those exposed under 100 *μ*g/L since 1995 (Table 4). However, this effect was not observed among those exposed since 1990 (Table 3) and was also not supported in the continuous analysis presented in Table 2 where no trend was evident.

### 3.4. Other Factors Associated with Mortality

The relationship between mortality rates and demographic and health characteristics among all residents are shown in Table 5. Lower education level was associated with increased mortality with a 27.5% increase in mortality rate with each lower completed education level. Heart disease mortality was strongly associated with farm work (IRR=3.31, p*<*0.001). Cancer mortality was strongly associated with smoking (IRR=2.47, p*<*0.001) and lower education level (IRR=1.76, p*<*0.001).

## 4. Discussion

Despite relatively moderate arsenic exposures, we observed an association between well-water arsenic exposure and heart disease mortality, cancer mortality, and all-cause mortality for the approximately eight year period from January 1, 1997 through December 1, 2004. The effect of arsenic on mortality was more pronounced among those exposed for longer periods of time, probably reflecting a lag time between exposure and observable effects on population mortality. The observation of an association between heart disease mortality and arsenic exposure among all residents at their current water source without any exposure lag period could reflect a shorter lag time between exposure to arsenic and the development of symptoms related to heart disease. Although we lacked sufficient cases to examine specific types of heart disease mortality and specific cancer mortality, the results we observed are consistent with previous studies linking cancer and heart disease mortality to arsenic exposure [3, 8, 23].

The cardiac effects of arsenic exposure are well known from acute high exposures in occupational [24] and medical settings [25], and intentional poisonings [26]. Other epidemiologic studies have observed associations between ischemic heart disease mortality [3, 8, 23], ischemic heart disease incidence [27], hypertensive heart disease mortality [28], hypertension [29], vascular disease mortality [30], carotid atherosclerosis [31], and increased blood pressure [32] and arsenic exposure.

Arsenic in drinking water is an established cause of skin, bladder and lung cancer [33]. Previous epidemiologic studies have shown associations with arsenic and elevated mortality from numerous cancers including and bladder [1, 2, 3],lung [1, 34, 35] and liver, kidney, prostate, stomach as well as other internal cancers [3, 4, 28, 34, 35, 36]. Our observations generally support these findings, providing evidence that after 10–20 years of arsenic exposure, there was an increase in all cancer mortality among residents of Shahai village related in a dose-response manner to arsenic exposure. The majority of cancers deaths were classified as lung, stomach, liver and esophagus. We observed only one death due to bladder cancer, and one due to skin cancer. Some authors have suggested a latency period between arsenic exposure and bladder cancer of 30 years or longer [37, 38]. Such a long latency period would make it difficult to observe an increase in bladder cancer mortality among Shahai residents who have been exposed at most about 20 years. The single skin cancer death was one of the most highly exposed residents of the village (525.2 *μ*g/L). Because the region is a rural area lacking a systematic method for classifying mortality, misdiagnoses, misclassification, and deaths due to other (competing) causes may also account for the low rate of bladder cancer mortality, and the misclassification of types of cancer mortality. Only 13 of the 76 cancer deaths were confirmed by pathology.

We cannot explain a decline in stroke mortality among respondents exposed to 5.1–20 *μ*g/L and 20.1–100 *μ*g/L among those exposed since 1995, but such a negative association between stroke mortality and arsenic exposure was not supported by the other analyses we conducted and presented. In contrast, studies of chronically exposed populations in Taiwan [4, 39], observed a positive association between arsenic exposure and cerebrovascular disease. An earlier study in Taiwan [35] and studies in occupationally exposed cohorts [40, 41] also failed to observe an association between cerebrovascular disease and arsenic exposure.

Our reliance on self-report and the lack of sophisticated diagnostic facilities may have lead to misclassification of cause of death, even for the broad categories of death we examined. To account for this possibility, we examined all-cause mortality, which would be unaffected by such misclassification, and would be accurately recalled by immediate family members. Despite the limitations resulting from self-report and potential misclassification, this study had significant strengths. The natural range in arsenic exposure in the village allowed us to compare highly exposed and less exposed residents within the same village for analysis. Different villages, regions, or populations could differ from Shahai village in numerous ways, resulting in a biased comparison. Unlike studies using a strictly ecological design, we had access to individual-level information and were able to control for important potential confounding factors. We were unable to control for some important covariates such as body mass index, diet and exercise. Although it is not clear how this may have affected our results, as we noted above, residents from this area are generally in good health and are predominantly from a single ethnic group (Han). A previous study found a low range of BMI in the region and found no direct association between BMI and prolonged QT interval [42].

It is unlikely that exposure to other sources of arsenic or other water sources affected our study results. The vast majority of residents rely solely on their household well for water. This well water is likely the main source of arsenic exposure in this region. There is no evidence of other major sources of arsenic exposure, for example, from indoor coal combustion or from pesticide applications.

We assumed that measured arsenic exposure at the water source in 2004 has remained relatively stable for the past 5, 10, 15 and 20 years. Based on limited data from 57 wells measured for arsenic in 1992 [43], we observed a good correlation to these same wells measured during this study (Pearsons r=0.76, p<0.0005). If arsenic changed substantially and the change was independently associated with mortality or morbidity due to other factors, our results may be biased with the direction of the bias difficult to predict. It seems more likely that any change in arsenic over time would be randomly associated with future mortality rates and such random misclassification would tend to attenuate our results toward no observable (null) effect. The results we observed would therefore be underestimates of the actual effect of arsenic on mortality.

We did not observe enough mortality to present a detailed categorical assessment of arsenic or an analysis of potential threshold effect points, and in the categorical analysis most results failed to achieve statistical significance. Without a large number of cases, valuable information can be lost by categorization, therefore we rely primarily on the continuous dose-response assessment for our inference and conclusions. Future studies should attempt to include large numbers of cases so a more detailed categorical, non-linear and threshold analysis can be reliably conducted.

In summary, for the period between January 1, 1997 and December 1, 2004, heart disease mortality was associated with arsenic levels in well water. Cancer mortality and all-cause mortality were also associated with arsenic levels in well water among those exposed at least 10–20 years. Cerebrovascular mortality was not associated with well-water arsenic exposure. This is the first study to document increased arsenic-associated mortality in the Bayingnormen Inner Mongolia region of China.

## Figures and Tables

**Table 1.** Characteristics of 572 deceased residents of Shahai village, Inner Mongolia.

	No.	%
Sex		
Male	327	57.2%
Female	245	42.8%
Total	572	100.0%

Age category		
0–5	6	1.1%
6–10	5	0.9%
11–15	1	0.2%
16–20	6	1.1%
21–30	11	1.9%
31–40	14	2.5%
41–50	34	6.0%
51–60	66	11.6%
61–70	144	25.3%
71–80	191	33.5%
Over 80	92	16.1%
Total	570	100.0%

Race/Ethnicity		
Han	571	99.8%
Mongol	1	0.2%
Total	572	100.0%

Occupation		
Agriculture	498	89.6%
Industry	2	0.4%
Professional	6	1.1%
Teacher	7	1.3%
Student	6	1.1%
Service	2	0.4%
Not employed	24	4.3%
Other	11	2.0%
Total	556	100.0%

Smoking		
Current smoker	261	46.4%
Ex-smoker	63	11.2%
Never smoked	239	42.5%
Total	563	100.0%

Education		
College	1	0.2%
High school	17	3.0%
Middle school	60	10.5%
Primary school	81	14.2%
Some primary or none	410	72.1%
Total	569	100.0%

Household income (Yuan)		
0–999	30	5.2%
1000–9999	391	68.4%
10000 or more	151	26.4%
Total	572	100.0%
Alcohol drinking		
Never drinker	381	67.8%
Ever drinker	181	32.2%
Total	562	100.0%
Years exposed to current water source		
Under 1	2	0.4%
1–10	247	43.4%
11–20	262	46.0%
21–30	58	10.2%
Total	569	100.0%
Well water arsenic (*μ*g/L)		
0–5	176	31.3%
5.1–20	102	18.1%
20.1–100	234	41.6%
100.1–300	42	7.5%
Over 300	8	1.4%
Total	562	100.0%
Well source		
Community well or other	145	25.5%
Hand pump well	424	74.5%
Total	569	100.0%

**Table 2.** Adjusted incidence rate ratios for mortality between January 1, 1997 and December 1, 2004 and arsenic exposure for all residents of Shahai village, and residents exposed to the same water source before 2000, 1995, 1990 and 1985.

All subjects				
	All-cause mortality IRR^1^/95% CI/p	Heart disease mortality IRR^1^/95% CI/p	Cancer mortality IRR^1^/95% CI/p	Stroke mortality IRR^1^/95% CI/p
Arsenic				
50 *μ*g/L increase	1.02	1.12*	1.07	0.82
	0.94,1.11	1.01,1.23	0.89,1.28	0.65,1.03
	0.616	0.034	0.464	0.086
Number	12600	12600	12600	12600
Exposed since before 2000				
	All-cause mortality IRR^1^/95% CI/p	Heart disease mortality IRR^1^/95% CI/p	Cancer mortality IRR^1^/95% CI/p	Stroke mortality IRR^1^/95% CI/p

Arsenic				
50 *μ*g/L increase	1.04	1.13*	1.09	0.82
	0.95,1.13	1.02,1.26	0.92,1.30	0.64,1.05
	0.394	0.016	0.305	0.109
Number	8967	8967	8967	8967
Exposed since before 1995				
	All-cause mortality IRR^1^/95% CI/p	Heart disease mortality IRR^1^/95% CI/p	Cancer mortality IRR^1^/95% CI/p	Stroke mortality IRR^1^/95% CI/p

Arsenic				
50 *μ*g/L increase	1.09	1.19**	1.18*	0.86
	0.99,1.19	1.05,1.33	1.00,1.40	0.67,1.12
	0.084	0.005	0.048	0.266
Number	6394	6394	6394	6394
Exposed since before 1990				
	All-cause mortality IRR^1^/95% CI/p	Heart disease mortality IRR^1^/95% CI/p	Cancer mortality IRR^1^/95% CI/p	Stroke mortality IRR^1^/95% CI/p

Arsenic				
50 *μ*g/L increase	1.12*	1.24***	1.20	0.88
	1.01,1.23	1.10,1.41	1.00,1.44	.68,1.15
	0.027	0.001	0.056	0.356
Number	3213	3213	3213	3213
Exposed since before 1985				
	All-cause mortality IRR^1^/95% CI/p	Heart disease mortality IRR^1^/95% CI/p	Cancer mortality IRR^1^/95% CI/p	Stroke mortality IRR^1^/95% CI/p

Arsenic				
50 *μ*g/L increase	1.15**	1.22*	1.22*	0.98
	1.03,1.27	1.03,1.44	1.03,1.45	0.76,1.26
	0.009	0.021	0.023	0.866
Number	1775	1775	1775	1775

1: Incidence rate ratio adjusted for age, sex, education, smoking, drinking, and farm work

* p*<*0.05,

** p*<*0.01,

*** p*<*0.001

**Table 3.** Crude death rates by arsenic exposure category among residents exposed since before 1990.

Arsenic (*μg/L*)	All deaths	Rate (per 100,000)	Adjusted IRR^1^	95% CI	p

0–5	115	1218.05	ref	ref	ref
5.1–20	49	985.49	0.87	0.62–1.22	0.413
20.1–100	86	1181.58	0.92	0.70–1.22	0.573
100.1–300	31	1459.02	1.05	0.71–1.54	0.815
Over 300	6	3599.29	3.39	1.32–8.69	0.011

Arsenic (*μg/L*)	Heart disease	Rate (per 100,000)	Adjusted IRR^1^	95% CI	p

0–5	36	381.3	ref	ref	ref
5.1–20	12	241.35	0.75	0.37–1.51	0.418
20.1–100	37	508.36	1.28	0.79–2.07	0.315
100.1–300	15	705.98	1.60	0.87–2.95	0.132
Over 300	2	1199.77	5.08	1.45–17.81	0.011

Arsenic (*μg/L*)	Cancer	Rate (per 100,000)	Adjusted IRR^1^	95% CI	p

0–5	11	116.51	ref	ref	ref
5.1–20	11	221.23	2.11	0.92–4.85	0.078
20.1–100	12	164.87	1.40	0.60–3.26	0.438
100.1–300	4	188.26	1.42	0.45–4.46	0.545
Over 300	1	599.89	6.25	1.08–36.22	0.041

Arsenic (*μg/L*)	Stroke	Rate (per 100,000)	Adjusted IRR^1^	95% CI	p

0–5	40	423.67	ref	ref	ref
5.1–20	13	261.46	0.62	0.33–1.18	0.147
20.1–100	20	274.79	0.65	0.38–1.12	0.118
100.1–300	6	282.39	0.58	0.26–1.29	0.181
Over 300	1	599.88	1.64	0.31–8.77	0.565

1: Incidence rate ratio adjusted for age, sex education, smoking, alcohol use, farm work

**Table 4.** Crude death rates by arsenic exposure category among residents exposed since before 1995.

Arsenic (*μg/L*)	All deaths	Rate (per 100,000)	Adjusted IRR^1^	95% CI	p

0–5	146	997.55	ref	ref	ref
5.1–20	79	873.26	0.89	0.68–1.17	0.401
20.1–100	179	837.75	0.84	0.68–1.06	0.139
100.1–300	37	1116.73	1.00	0.70–1.42	1.000
Over 300	6	2408.52	2.28	0.75–6.95	0.146

Arsenic (*μg/L*)	Heart disease	Rate (per 100,000)	Adjusted IRR^1^	p	

0–5	44	300.63	ref	ref	ref
5.1–20	26	287.4	1.07	0.64–1.78	0.810
20.1–100	72	336.97	1.22	0.82–1.82	0.317
100.1–300	17	513.09	1.55	0.88–2.73	0.125
Over 300	2	802.83	2.47	0.50–12.18	0.265

Arsenic (*μg/L*)	Cancer	Rate (per 100,000)	Adjusted IRR^1^	95% CI	p

0–5	15	102.49	ref	ref	ref
5.1–20	14	154.75	1.62	0.79–3.33	0.191
20.1–100	24	112.32	1.13	0.59–2.19	0.708
100.1–300	6	181.09	1.50	0.58–3.85	0.398
Over 300	1	401.42	3.99	0.59–27.17	0.157

Arsenic (*μg/L*)	Stroke	Rate (per 100,000)	Adjusted IRR^1^	95% CI	p

0–5	53	362.13	ref	ref	ref
5.1–20	16	176.86	0.47	0.27–0.84	0.010
20.1–100	41	191.89	0.51	0.34–0.79	0.002
100.1–300	7	211.27	0.52	0.25–1.10	0.087
Over 300	1	401.41	1.02	0.16–6.71	0.980

1: Incidence rate ratio adjusted for age, sex education, smoking, alcohol use, farm work

**Table 5.** Poisson regression model results for mortality between January 1, 1997 and December 1, 2004 and arsenic exposure for all residents of Shahai village.

All subjects				
	All-cause mortality IRR^1^/95% CI/p	Heart disease mortality IRR^1^/95% CI/p	Cancer mortality IRR^1^/95% CI/p	Stroke mortality IRR^1^/95% CI/p
Arsenic				
50 *μ*g/L increase	1.02	1.12*	1.07	0.82
	0.94,1.11	1.01,1.23	0.89,1.28	0.65,1.03
	0.616	0.034	0.464	0.086
Age				
	1.09***	1.14***	1.05***	1.11***
	1.08,1.10	1.12,1.15	1.03,1.07	1.09,1.13
	0.000	0.000	0.000	0.000
Sex				
(female vs male)	0.83	1.18	0.63	0.85
	0.67,1.02	0.86,1.62	0.38,1.07	0.55,1.29
	0.073	0.300	0.085	0.440
Education^2^				
	1.27**	1.03	1.75**	1.20
	1.09,1.49	0.81,1.30	1.20,2.57	0.87,1.66
	0.003	0.832	0.004	0.267
Current smoker				
(yes vs. no)	1.17	1.33	2.47***	1.13
	0.97,1.42	0.96,1.84	1.53,3.99	0.76,1.68
	0.101	0.085	0.000	0.551
Alcohol use				
(yes vs. no)	1.15	0.96	1.32	0.97
	0.94,1.40	0.67,1.36	0.79,2.20	0.64,1.47
	0.178	0.802	0.284	0.882
Farmer				
(yes vs. no)	1.27	3.31***	1.72	1.29
	0.93,1.73	1.83,5.98	0.71,4.18	0.74,2.26
	0.132	0.000	0.233	0.375
N	12600	12600	12600	12600

1: 1: Incidence rate ratio adjusted for age, sex, education, smoking, drinking, and farm work

* p*<*0.05,

** p*<*0.01,

*** p*<*0.001

2: IRR represents the effect of each lower completed education level (1=college, 2-high school, 3=middle school, 4=primary school, 5=less than primary school)

## References

[1] Smith AH, Goycolea M, Haque R, Biggs ML (1998). Marked increase in bladder and lung cancer mortality in a region of Northern Chile due to arsenic in drinking water. Am. J. Epidemiol.

[2] Hopenhayn-Rich C, Biggs ML, Fuchs A, Bergoglio R, Tello EE, Nicolli H, Smith AH (1996). Bladder cancer mortality associated with arsenic in drinking water in Argentina. Epidemiology.

[3] Chen CJ, Chuang YC, Lin TM, Wu HY (1985). Malignant neoplasms among residents of a blackfoot disease-endemic area in Taiwan: high-arsenic artesian well water and cancers. Cancer Res.

[4] Tsai SM, Wang TN, Ko YC (1999). Mortality for certain diseases in areas with high levels of arsenic in drinking water. Arch. Environ. Health.

[5] Otto D, He L, Xia Y, Li Y, Wu K, Ning Z, Zhao B, Hudnell HK, Kwok R, Mumford J, Geller A, Wade T (2006). Neurosensory effects of chronic exposure to arsenic via drinking water in Inner Mongolia: II. vibrotactile and visual function. J. Water Health.

[6] Li Y, Xia Y, He L, Ning Z, Wu K, Zhao B, Le XC, Kwok R, Schmitt M, Wade T, Mumford J, Otto D (2006). Neurosensory effects of chronic exposure to arsenic via drinking water in Inner Mongolia: I. Signs, symptoms and pinprick testing. J. Water Health.

[7] Chen CJ, Hsueh YM, Lai MS, Shyu MP, Chen SY, Wu MM, Kuo TL, Tai TY (1995). Increased prevalence of hypertension and long-term arsenic exposure. Hypertension.

[8] Chang CC, Ho SC, Tsai SS, Yang CY (2004). Ischemic heart disease mortality reduction in an arseniasis-endemic area in southwestern Taiwan after a switch in the tap-water supply system. J. Toxicol. Environ. Health A.

[9] Ma H, Xia Y, Wu K (1999). Human exposure to arsenic and health effects in Bayingnormen, Inner Mongolia. Arsenic Exposure and Health Effects.

[10] Yu G, Sun D, Zheng Y (2007). Health effects of exposure to natural arsenic in groundwater and coal in China: an overview of occurrence. Environ. Health Perspect.

[11] Guo X, Fujino Y, Ye X, Liu J, Yoshimura T (2006). Association between multi-level inorganic arsenic exposure from drinking water and skin lesions in China. Int. J. Environ. Res. Public Health.

[12] Guo X, Fujino Y, Kaneko S, Wu K, Xia Y, Yoshimura T (2001). Arsenic contamination of ground-water and prevalence of arsenical dermatosis in the hetao plain area, inner mongolia, china. Mol. Cell Biochem.

[13] Mo J, Xia Y, Wade TJ, Schmitt M, Le XC, Dang R, Mumford JL (2006). Chronic arsenic exposure and oxidative stress: OGG1 expression and arsenic exposure, nail selenium, and skin hyperkeratosis in Inner Mongolia. Environ. Health Perspect.

[14] (2006). International classification of diseases, 10th revision.

[15] Hoyert D, Heron M, Murphy S, Kung H (2006). Deaths: Final data for 2003. National vital statistics reports; Technical report.

[16] Gong Z, Lu X, Watta C, Wena B, Hea B, Mumford J, Ning Z, Xia Y, Le XC (2006). Speciation analysis of arsenic in groundwater from Inner Mongolia with an emphasis on acid-leachable particulate arsenic. Anal. Chim. Acta.

[17] Selvin S (1996). Statistical Analysis of Epidemiological Data.

[18] Williams RL (2000). A note on robust variance estimation for cluster-correlated data. Biometrics.

[19] StataCorp LP (2006). Stata/SE 92 for Windows.

[20] Ahmad OB, Boschi-Pinto C, Lopez AD, Murray CJ, Lozano R, Inoue M (2001). Age standardization of rates: A new WHO standard Technical Report GPE Discussion Paper Series: No 31.

[21] World Health Organization (2004). Estimates of death rates for 2002 by cause for WHO Member States.

[22] Liu Z, Lobdell DT, Myers SL, He L, Yang M, Kwok RK, Mumford JL, Mendola P (2007). Pregnancy and perinatal health in Inner Mongolia, China, 1996–1999. Int. J. Gynaecol. Obstet.

[23] Chen CJ, Chiou HY, Chiang MH, Lin LJ, Tai TY (1996). Dose-response relationship between ischemic heart disease mortality and long-term arsenic exposure. Arterioscler. Thromb. Vasc. Biol.

[24] Axelson O, Dahlgren E, Jansson CD, Rehnlund SO (1978). Arsenic exposure and mortality: a case-referent study from a Swedish copper smelter. Br. J. Ind. Med.

[25] Westervelt P, Brown RA, Adkins DR, Khoury H, Curtin P, Hurd D, Luger SM, Ma MK, Ley TJ, DiPersio JF (2001). Sudden death among patients with acute promyelocytic leukemia treated with arsenic trioxide. Blood.

[26] St Petery J, Gross C, Victorica BE (1970). Ventricular fibrillation caused by arsenic poisoning. Am. J. Dis. Child.

[27] Tseng CH (2003). Abnormal current perception thresholds measured by neurometer among residents in blackfoot disease-hyperendemic villages in Taiwan. Toxicol. Lett.

[28] Lewis DR, Southwick JW, Ouellet-Hellstrom R, Rench J, Calderon RL (1999). Drinking water arsenic in Utah: A cohort mortality study. Environ. Health Perspect.

[29] Rahman M, Tondel M, Ahmad SA, Chowdhury IA, Faruquee MH, Axelson O (1999). Hypertension and arsenic exposure in Bangladesh. Hypertension.

[30] Engel RR, Hopenhayn-Rich C, Receveur O, Smith AH (1994). Vascular effects of chronic arsenic exposure: a review. Epidemiol. Rev.

[31] Wang CH, Jeng JS, Yip PK, Chen CL, Hsu LI, Hsueh YM, Chiou HY, Wu MM, Chen CJ (2002). Biological gradient between long-term arsenic exposure and carotid atherosclerosis. Circulation.

[32] Kwok RK, Mendola P, Liu ZY, Savitz DA, Heiss G, Ling HL, Xia Y, Lobdell D, Zeng D, Thorp JM, Creason JP, Mumford JL (2007). Drinking water arsenic exposure and blood pressure in healthy women of reproductive age in Inner Mongolia, China. Toxicol. Appl. Pharmacol.

[33] (2004). Some drinking-water disinfectants and contaminants, including arsenic.

[34] Chen CJ, Wang CJ (1990). Ecological correlation between arsenic level in well water and age-adjusted mortality from malignant neoplasms. Cancer Res.

[35] Wu MM, Kuo TL, Hwang YH, Chen CJ (1989). Dose-response relation between arsenic concentration in well water and mortality from cancers and vascular diseases. Am. J. Epidemiol.

[36] Hopenhayn-Rich C, Biggs ML, Smith AH (1998). Lung and kidney cancer mortality associated with arsenic in drinking water in Cordoba, Argentina. Int. J. Epidemiol.

[37] Steinmaus C, Yuan Y, Bates MN, Smith AH (2003). Case-control study of bladder cancer and drinking water arsenic in the western United States. Am. J. Epidemiol.

[38] Bates MN, Smith AH, Cantor KP (1995). Case-control study of bladder cancer and arsenic in drinking water. Am. J. Epidemiol.

[39] Chiou HY, Huang WI, Su CL, Chang SF, Hsu YH, Chen CJ (1997). Dose-response relationship between prevalence of cerebrovascular disease and ingested inorganic arsenic. Stroke.

[40] Hertz-Picciotto I, Arrighi HM, Hu SW (2000). Does arsenic exposure increase the risk for circulatory disease?. Am. J. Epidemiol.

[41] Jarup L, Pershagen G, Wall S (1989). Cumulative arsenic exposure and lung cancer in smelter workers: a dose-response study. Am. J. Ind. Med.

[42] Mumford JL, Wu K, Xia Y, Kwok R, Yang Z, Foster J, Sanders WE (2007). Chronic arsenic exposure and cardiac repolarization abnormalities with qt interval prolongation in a population-based study. Environ. Health Perspect.

[43] Ning Z, Lobdell DT, Kwok RK, Liu Z, Zhang S, Ma C, Riediker M, Mumford JL (2007). Residential exposure to drinking water arsenic in Inner Mongolia, China. Toxicol. Appl. Pharmacol.

